# Age-Dependent Variations in Kawasaki Disease Incidence in Japan

**DOI:** 10.1001/jamanetworkopen.2023.55001

**Published:** 2024-02-06

**Authors:** Laurel L. DeHaan, Charles D. Copeland, Jennifer A. Burney, Yosikazu Nakamura, Mayumi Yashiro, Chisato Shimizu, Koichi Miyata, Jane C. Burns, Daniel R. Cayan

**Affiliations:** 1 Scripps Institution of Oceanography, University of California San Diego; 2 School of Global Policy and Strategy, University of California San Diego; 3 Department of Pediatrics, School of Medicine, University of California San Diego; 4 Rady Children’s Hospital, San Diego, California; 5 Department Public of Health, Jichi Medical University, Tochigi, Japan

## Abstract

**Importance:**

The etiology of Kawasaki disease (KD) remains elusive, with immunologic and epidemiologic data suggesting different triggers in individuals who are genetically susceptible. KD remains the most common cause of acquired heart disease in pediatric patients, and Japan is the country of highest incidence, with an increasing number of cases.

**Objective:**

To investigate whether an analysis of the epidemiologic KD record in Japan stratified by age and prefecture (subregion) may yield new clues regarding mechanisms of exposure to etiologic agents associated with KD.

**Design, Setting, and Participants:**

This cross-sectional study was conducted using a dataset of patients with KD with detailed information on location and age at onset created through nationwide surveys of hospitals caring for pediatric patients with KD throughout Japan. Pediatric patients hospitalized in Japan for KD from 1970 to 2020 were included. Data were analyzed from January 2022 to January 2024.

**Exposure:**

Pediatric patients with KD.

**Main Outcomes and Measures:**

The KD dataset was analyzed by patient age (infants [aged <6 months], toddlers [aged 6 to <24 months], children aged 2 years [aged 24 to <36 months], and children and adolescents aged 3 years or older [aged ≥36 months]), with investigations of seasonal cycles, interannual variations, and correlations across regions.

**Results:**

Among 422 528 pediatric patients (243 803 males [57.7%] and 178 732 females [42.3%]; median [IQR] age, 23.69 [11.96-42.65] months), infants, toddlers, and patients aged 3 years or older exhibited different rates of increase in KD incidence, seasonality, and degrees of coherence of seasonality across prefectures. Although the mean (SD) incidence of KD among infants remained relatively stable over the past 30 years compared with older patients (1.00 [0.07] in 1987-1992 to 2.05 [0.11] in 2011-2016), the mean (SD) incidence rate for children and adolescents aged 3 years or older increased 5.2-fold, from 1.00 (0.08) in 1987 to 1992 to 5.17 (0.46) in 2014 to 2019. Patients aged 3 years or older saw a reduction in mean (SD) incidence, from peaks of 5.71 (0.01) in October 2014 through June 2015 and July 2018 through March 2019 to 4.69 (0.11) in 2016 to 2017 (17.8% reduction) not seen in younger children. The seasonal cycle varied by age group; for example, mean (SD) incidence peaked in July and August (5.63 [0.07] cases/100 000 individuals) for infants and in December and January (4.67 [0.13] cases/100 000 individuals) for toddlers. Mean (SD) incidence changed dramatically for toddlers beginning in the early 2010s; for example, the normalized mean (SD) incidence among toddlers for October was 0.74 (0.03) in 1992 to 1995 and 1.10 (0.01) in 2016 to 2019. Across Japan, the seasonal cycle of KD incidence of older children and adolescents exhibited mean (SD) correlation coefficients between prefectures as high as 0.78 (0.14) for prefecture 14 among patients aged 3 years or older, while that of infants was much less (highest mean [SD] correlation coefficient, 0.43 [0.23]).

**Conclusions and Relevance:**

This study found distinct temporal signatures and changing spatial consistency of KD incidence across age groups, suggesting different age-related mechanisms of exposure. Some results suggested that social factors may modulate exposure to etiologic agents of KD; however, the increase in KD incidence in older children coupled with the correlation across prefectures of KD incidence suggest that the intensity of an environmental exposure that triggers KD in this age group may have increased over time.

## Key Points

**Question:**

Does the epidemiologic Kawasaki disease (KD) record in Japan yield new clues regarding the mechanism of disease transmission when stratified by age?

**Findings:**

In this cross-sectional study of 422 528 pediatric patients with KD in Japan, there were differences in KD incidence by age group and region, including the rate of increase over the past 3 decades, seasonal cycle, and correlation of the seasonal cycle between prefectures.

**Meaning:**

This study found distinct seasonality and differences in correlations between prefectures of the seasonal cycle of KD incidence across age groups, suggesting different age-related forms of exposure that may need to be accounted for in explanations of KD etiology.

## Teaser

This cross-sectional study investigates rates of Kawasaki disease among pediatric patients in Japan by age, including analyses of seasonal cycles, interannual variations, and correlations across regions.

## Introduction

Despite 5 decades of research, the etiology of Kawasaki disease (KD), the leading cause of acquired heart disease in children and adolescents, remains a mystery. Tomisaku Kawasaki first described the condition in a landmark publication^1^ reporting 50 pediatric patients with fever, rash, mucocutaneous findings, and lymphadenopathy. Without treatment, the acute phase of the illness can last for weeks and then resolve spontaneously. However, 25% of untreated pediatric patients will develop aneurysms of the coronary arteries that may lead to myocardial infarction and death.^2^ Treatment with intravenous immunoglobulin within days after the onset of fever reduces the risk of cardiac complications.^3^ However, identification of the triggers for KD would be transformative for patient care and would surely lead to improved diagnostics and more specific treatments.

Most authorities believe that KD was a new pediatric disease that emerged in Japan after World War II. Patients meeting the clinical case definition were rare in the 1950s.^4^ Nationwide epidemics in 1979, 1982, and 1986 were consistent with new widespread exposure in a highly susceptible population.^5^ Subsequent research has revealed an important role for genetic variants that are associated with susceptibility to KD.^6^ Japan remains the country of highest incidence, although cases are now diagnosed worldwide.^7^ While the etiology remains unknown, a complex interplay of host genetics and environmental factors is suspected.

The epidemiology team at Jichi University has meticulously curated a nationwide survey of KD in Japan since 1970, which has fueled decades of research on KD. Basic features of KD epidemiology in Japan that have been gleaned from this source include the following: incidence with a male-to-female ratio of 1.5:1, 85% of patients younger than age 5 years, an increase in the total number of cases in Japan over time, peak incidence at age 9 months, and a low recurrence rate of 3% to 4%.^8,9,10^ Further analyses using the database have identified an overall seasonal structure of KD incidence and spatiotemporal clustering, with similar characteristics noted in North America, the UK, and Western Europe.^11,12^ A previous analysis of 127 398 Japanese patients with KD explored age-related differences in KD incidence and seasonality.^13^ In this study, we add to this investigation by conducting an analysis over a longer period at higher spatial and temporal resolution, enabling a more robust study of age-dependent seasonality and changes in seasonality over decades, as well as spatial coherence of KD incidence.

## Methods

The nationwide KD survey used in this cross-sectional study was approved by the Bioethical Committee for Epidemiologic Research, Jichi Medical University, Japan.^14^ This study was reviewed and a waiver of consent approved by the University of California San Diego Institutional Review Board, which does not require informed consent for any of our activities. The study followed the Strengthening the Reporting of Observational Studies in Epidemiology (STROBE) reporting guideline.

### Statistical Analysis

**Figure 1.**
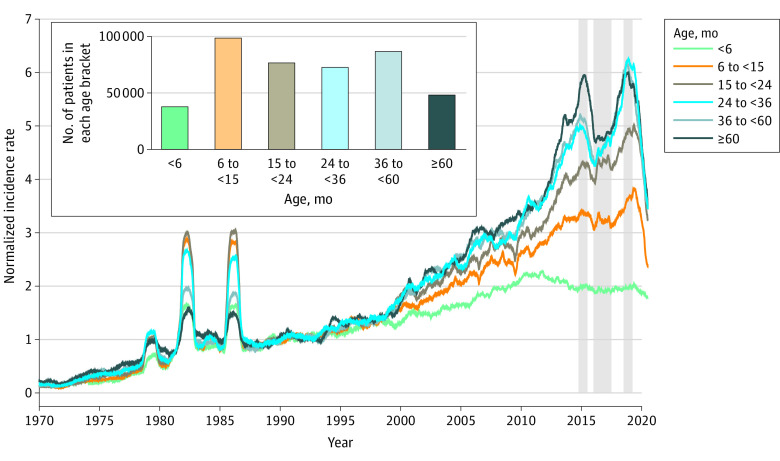
Normalized Time Series of Kawasaki Disease Incidence Rate by Age Group Time series are presented of Kawasaki disease incidence for each age group, normalized by dividing by the mean number of cases in 1987 to 1992 for each age bracket, based on daily smoothed data. To account for the decreasing birth rate in Japan, the incidence rate was computed relative to the population of children and adolescents aged 0 to 14 years for each year. The inset shows the number of patients in each age bracket for the 1970 to 2020 record. The 3 shaded areas in 2014 to 2019 indicate the decrease in cases (middle shaded area) and peaks on either side. For comparison, the time series that is not normalized by the 1987 to 1992 base period is shown in eFigure 1 and eAppendix 1 in Supplement 1.

We hypothesized that different age groups of patients with KD would exhibit differences in KD incidence, seasonal patterns, and spatiotemporal correlations in seasonality across Japan’s 47 prefectures (subregions). To test these hypotheses, we used data compiled from 26 nationwide surveys of patients with KD in Japan, which provided the date of birth, date of onset, and home prefecture of 422 528 patients between 1970 and 2020. Starting in 1970, the survey was conducted every 2 years by questionnaires sent to pediatricians at hospitals with more than 100 beds and at specialized pediatric hospitals. The response rate was more than 70% (eg, 1444 of 1881 facilities [76.8%] reported in the 2015-2016 survey) for the last 2 decades, and most nonresponding hospitals had few or no patients with KD.^15^ The diagnosis of patients with KD has been consistent over the period of record based on guidelines created by the Japan Kawasaki Disease Research Committee. Using these data, a 50-year time series of KD incidence rate was computed by counting the number of patients on each date of onset in the record and smoothing that count with a 365-day running mean (the mean was found for the value of each day with the 182 previous days and 182 subsequent days). We smoothed the data with a 365-day running mean to remove the seasonal cycle from the result. To account for the decreasing birth rate in Japan, the incidence rate was computed relative to the population of individuals aged 0 to 14 years from the e-Stat Portal Site of the Government Statistics of Japan.^16^ Time-series values were then normalized by dividing incidence rates in each of 6 age brackets (<6 months, 6 to <15 months, 15 to <24 months, 24 to <36 months, 36 to <60 months, and ≥60 months) by the mean number of patients in that age bracket between 1987 and 1992, when the total annual number of patients with KD in Japan was relatively constant (Figure 1). The result is the KD incidence rate for each age bracket relative to the incidence rate from 1987 to 1992. For each age bracket, we performed 2-tailed *t* tests to compare recent mean incidence rates with those within the stable, 1987 to 1992 postepidemic period and the 2016 to 2017 period of low incidence, with mean rates of peaks on either side, wherein *P*-values < .05 were considered significant.

We further analyzed data by seasonal cycle using a subset of the Japanese nationwide surveys (1988-2019). The 1979, 1982, and 1986 KD epidemics and the COVID-19 pandemic greatly altered the incidence and seasonality of KD.^17,18^ Consequently, the record beginning in 1988 and ending in 2019 was used to avoid the influence of the KD epidemics and COVID-19 pandemic. For this analysis, we computed the seasonal cycle of KD incidence rate based on a time series smoothed with a 31-day running mean by finding the mean of the number of KD onsets for each day of the year between 1988 and 2019. We used a 31-day running mean to remove the day-to-day variation in the record. Unique seasonal cycles were computed for 4 age groups and Japan’s 47 prefectures. Age groups were characterized as infants (aged <6 months; 28 147 of 323 530 patients in 1988-2019 [8.7%]), toddlers (aged 6 to <24 months; 131 353 patients [40.6%]), children aged 2 years (aged 24 to <36 months; 56 294 patients [17.4%]), and children and adolescents aged 3 years or older (aged ≥36 months; 107 736 patients [33.3%]). The toddler group combines 2 age groups from Figure 1 based on the similar incidence patterns of those age groups, and likewise the ages 3 years and older group is a combination of 2 groups from Figure 1. In an analysis of changes in seasonal cycles over 1988 to 2019, incidence rates were normalized by dividing by the mean incidence rate for relevant subsets of years. To test for significant differences between seasonal cycles, we used a random sampling approach, as described in eFigure 2 in Supplement 1.

A third analysis was performed to investigate similarities of seasonal cycles among Japan’s 47 prefectures. A seasonal cycle for each prefecture was computed as described previously, and Pearson correlations of seasonal cycles were computed between each pair of prefectures to investigate coherence of KD incidence across regions. The mean of the resulting 46 correlations for each prefecture was then found to give a single value for each prefecture. Pairwise correlations greater than 0.59 were significant at 95%. All computations were performed in MATLAB version R2023a (Mathworks). Data were analyzed from January 2022 to January 2024.

## Results

Among 422 528 pediatric patients (243 803 males [57.7%] and 178 732 females [42.3%]; median [IQR] age, 23.69 [11.96-42.65] months), time series of KD incidence rates from 1970 to 2000 for 6 age groups showed that the well-known increase in KD incidence in Japan over time was evident in each age group to varying extents (Figure 1). However, the increase in normalized incidence rates differed among age groups, particularly between infants and children and adolescents aged 3 years and older. After the major KD epidemics in 1979, 1982, and 1986, each age group experienced a period of stable KD incidence until the late 1990s. Thereafter, from 2000 through 2019, incidence increased rapidly. For infants, mean (SD) KD incidence increased by a factor of 2.1 from the base period in 1987 to 1992 (1.00 [0.07]) to the peak years of 2011 to 2016 (2.05 [0.11]) (*P* < .001). In contrast, KD incidence for the children and adolescents aged 3 years and older (ie, the 2 oldest age groups) increased by a factor of 5.2 between the base period in 1987 to 1992 (1.00 [0.08]) and the peak years for this group, 2014 to 2019 (5.17 [0.46]) (*P* < .001). There were 2 marked decreases in KD incidence during this period. The first was in 2016 and 2017. For children and adolescents aged 3 years and older, the decrease, highlighted in the center gray box in Figure 1 (January 2016 through June 2017), was a reduction in the mean (SD) incidence from peaks of 5.71 (0.10) on either side (October 2014 through June 2015 and July 2018 through March 2019) to 4.69 (0.11) (*P* = .005), a 17.8% decrease. The reduction in mean (SD) incidence for children aged 6 to less than 24 months was from peaks of 4.02 (0.06) to 3.74 (0.10) (*P* = .02), and there was no significant reduction for infants. The second decline was a sharp decrease in cases in 2020 that coincided with the COVID-19 pandemic.^17^

**Figure 2.**
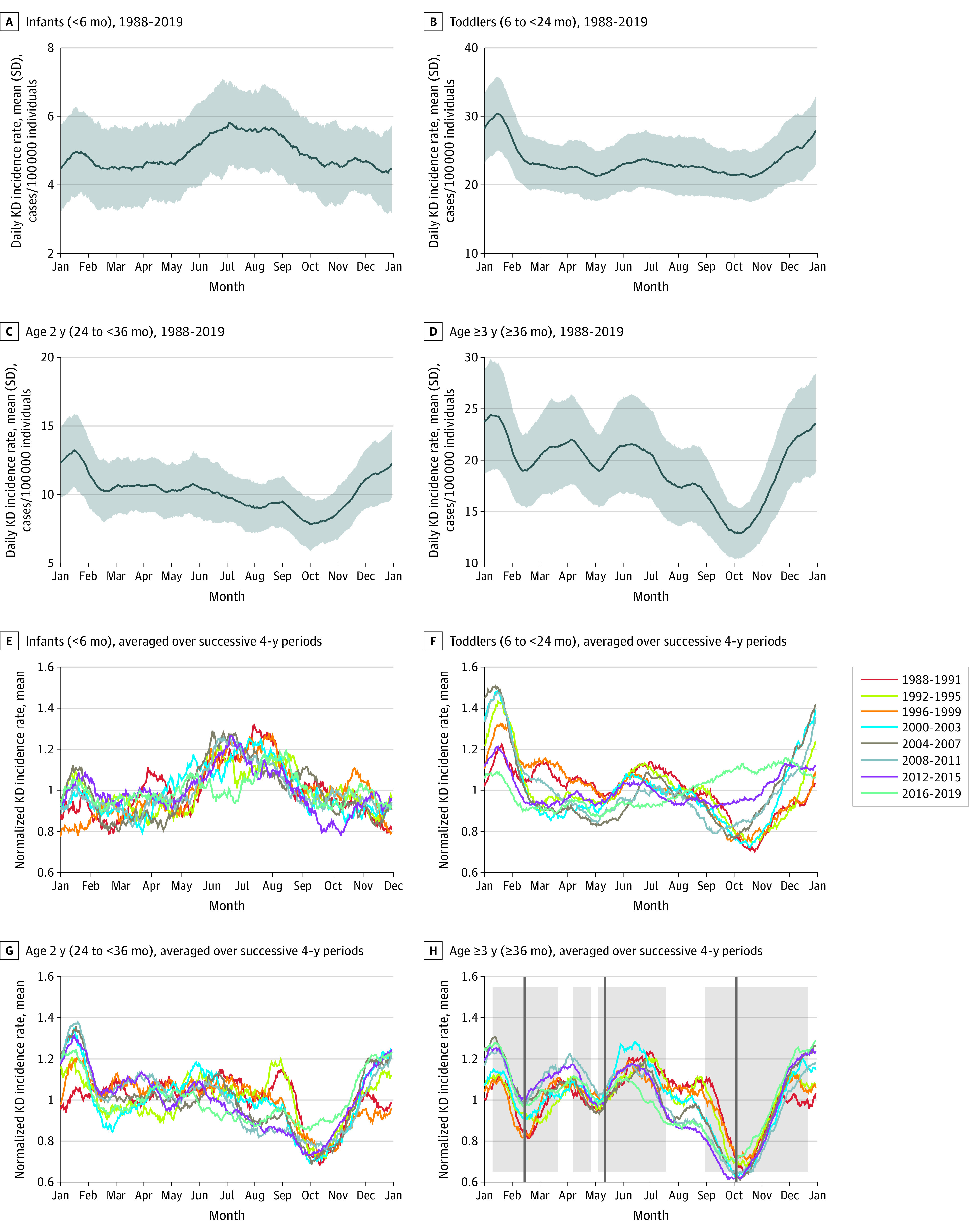
Seasonal Cycle of Kawasaki Disease (KD) Incidence Rate for 4 Age Groups The seasonal cycle of daily KD incidence rate per 100 000 individuals younger than age 14 years is presented for 4 age groups for the mean over 1988 to 2019 (A-D) and the mean over successive 4-year periods (E-H). Panels E to H were normalized by the mean incidence rate for the relevant subsets of years. The vertical axis differs for each panel in A to D. Shading in A to D shows the SD of the seasonal cycle between prefectures, with 1 SD above and below the mean. Shaded boxes on H delineate typical periods during which school is in session. Note that not all children between ages 36 and 72 months attend preschool, which has this school schedule. This schedule does not apply to children in day care or at home. Vertical lines on H mark 5 weeks after the start of the school session.

We analyzed seasonal cycles of KD incidence in each age bracket using a subset of the Japanese nationwide survey (1988-2019, which avoided the KD epidemics and COVID-19 pandemic) with 323 531 patients (186 353 males [57.6%] and 137 177 females [42.4%]; median [IQR] age, 24.26 [12.24-43.56] months). These long-term means (Figure 2) showed different seasonal cycles of KD incidence rates for each age group. Infants experienced a lower overall mean (SD) incidence than other age groups but nonetheless had a discernable peak in July and August of 5.63 (0.07) cases/100 000 individuals that was an increase of nearly 20% over the other 10 months (4.78 [0.31] cases/100 000 individuals, for a 17.8% increase). In contrast, toddlers (patients aged 6 to <24 months) experienced a seasonal peak in mean (SD) incidence in December and January (4.67 [0.13] cases/100 000 individuals), with relatively constant numbers from March through October. Children aged 2 years had a similar winter peak as toddlers but exhibited a pronounced decrease in October. Children and adolescents aged 3 years or older showed the most complex seasonal cycle. The pattern was similar to that of children aged 2 years, with a winter peak and an autumn nadir, but also included secondary peaks in April and June. The typical school calendar shown by the shaded boxes in Figure 2H highlights that the number of KD cases among children and adolescents aged 3 years or older decreased at the beginning of each school session and then increased after approximately 5 weeks of school attendance.

A more detailed analysis of long-term, 1988 to 2019 means (Figure 2A-D) revealed shifts and distortions of the overall seasonal cycle over time, shown by seasonal cycles of normalized KD incidence for successive 4-year periods (Figure 2E-H). Most larger differences exceeded the 95th or 99th percentile of statistical odds of random occurrence; for example, toddlers had significantly higher incidence in spring (April, May, and June) in 1988 to 1991 compared with other years (eFigure 2 and eAppendix 2 in Supplement 1). Infants, who exhibited the least amount of change over time (Figure 2E), had a summer peak that consistently occurred from the early 1990s until 2015. In 2016 to 2019, the summer peak disappeared. Incidence patterns among toddlers changed the most (Figure 2F). Toddlers experienced a January peak and an October nadir in the 1990s until the early 2000s, but their seasonal cycle shifted in 2012 to 2015 such that October was no longer a low point, and during 2016 to 2019, October became the peak in the seasonal cycle. The normalized mean (SD) incidence among toddlers for October was 0.74 (0.03) in 1992 to 1995 and 1.10 (0.01) in 2016 to 2019.

Children aged 2 years (Figure 2G) had a consistent January peak and October nadir until 2015. However, the October low disappeared during 2016 to 2019 as with toddlers. In comparison with toddlers, children and adolescents aged 3 years and older had a seasonal cycle structure (Figure 2H) that was consistent throughout the period. The October nadir occurred throughout the period, as did the January peak. Secondary features of KD incidence in children and adolescents aged 3 years and older were also consistent; peaks in April, June, and September occurred across decades.

**Figure 3.**
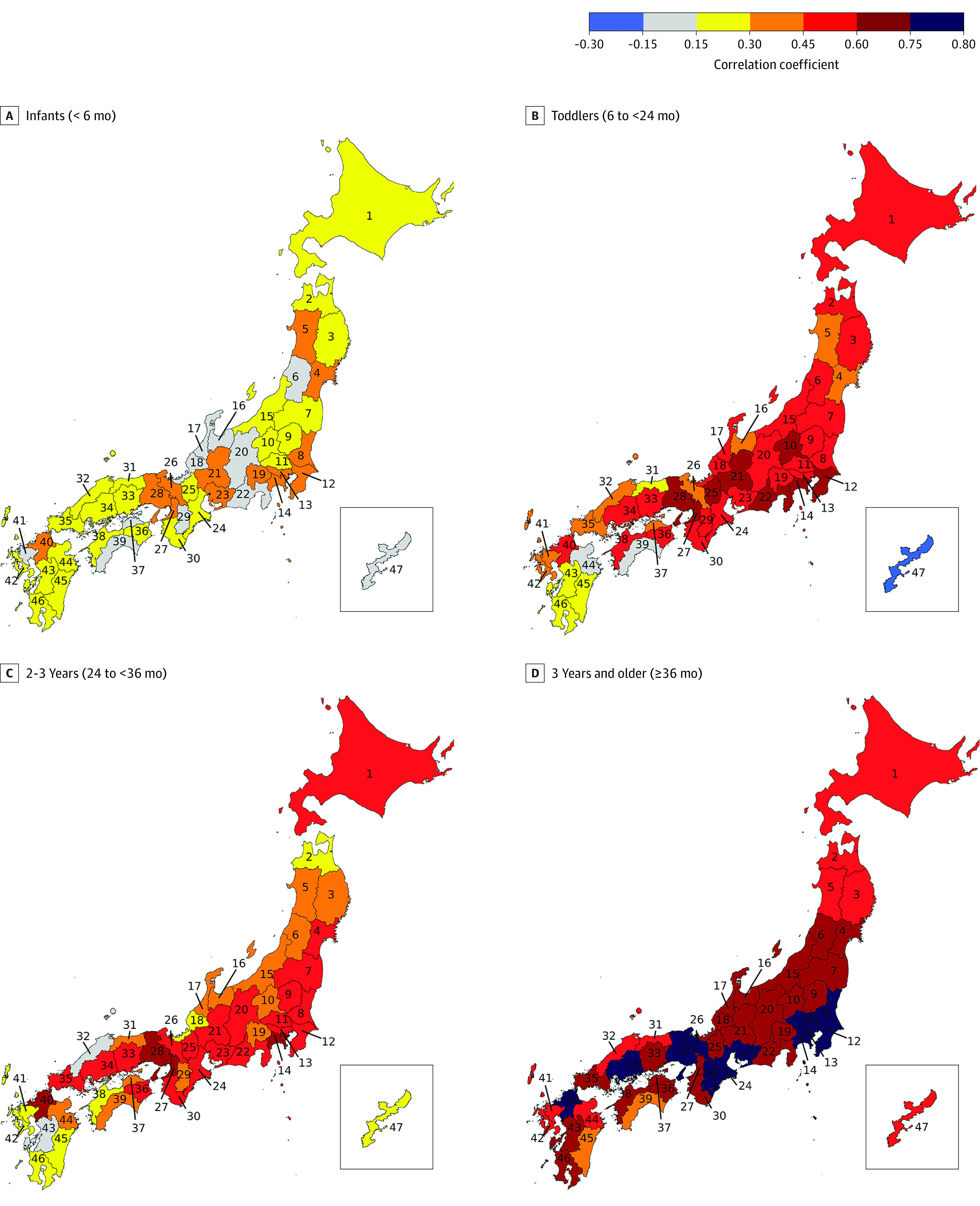
Correlations of Kawasaki Disease Seasonal Cycles Between Prefectures Mean correlations of Kawasaki disease seasonal cycles (1988-2019) between prefectures are presented for 4 age groups. Correlations greater than 0.59 are significant at 95%. The southern island of Okinawa is shown in the inset. Numbers within the map indicate prefecture identifiers.

To assess whether seasonal cycle varied spatially across Japan, the data record was disaggregated into prefectures and the seasonal cycle for each prefecture was correlated with that of every other prefecture (Figure 3; eFigure 3 and eAppendix 3 in Supplement 1). For infants, seasonal cycles of different prefectures showed essentially no correlation with other prefectures; mean (SD) correlation coefficients ranged from 0.02 (0.22) for prefecture 18 to 0.43 (0.23) for prefecture 27 (Figure 3A). Seasonal cycles for toddlers and children aged 2 years (Figure 3B and Figure 3C) exhibited higher correlations, with a few prefectures having mean correlations at greater than the 95% significance level. The lowest correlation coefficients for toddlers and children aged 2 years occurred in prefectures on the southern island of Kyushu. Seasonal cycles for children and adolescents aged 3 years and older (Figure 3D) exhibited the greatest spatial coherence, with more than 80% of prefecture pairs (38 of 47 pairs [80.9%]) having mean correlations at greater than the 95% significance level; for example, mean (SD) correlation coefficients were as high as 0.78 (0.14) for prefecture 14. Although age groups had different sample sizes, the large difference between the low correlation coefficients of infants and high correlation coefficients of children and adolescents aged 3 years and older across prefectures may reduce concerns about spurious correlations due to small sample size.

There was additional time-varying structure underlying these prefecture-level associations. In earlier years, correlation coefficients across prefectures for toddlers were higher than those for children and adolescents aged 3 years and older. However, in later years there was a decrease in correlation coefficients for toddlers while correlation coefficients for children and adolescents aged 3 years and older increased (eFigure 4 and eAppendix 4 in Supplement 1).

## Discussion

Although previous studies have described overall KD incidence in Japan, a sharper and more complex picture emerged when KD incidence was assessed by age groups in this cross-sectional study. Age-associated patterns may add new insights into the epidemiology of KD in Japan and are thus important clues to understanding the etiology of KD. Hypotheses regarding KD etiology must explain the distinctly different epidemiology between infants and older children and adolescents; changing seasonal cycle for toddlers; complex seasonal cycle for children and adolescents aged 3 years and older, with secondary peaks in the spring that aligned across prefectures; and 5-fold increase in incidence rates for older children and adolescents since 1990. These findings argue for both social factors modulating exposure, including person-to-person spread, as well environmental exposures that are increasing over time. The summary of these characteristics, along with the characteristics of previous studies, are listed in the Box.^4,10,11,14,17,18,19,20,21^

Box. **Characteristics of KD Incidence in Japan**

**Previous findings**

New disease after WWII (Shibuya et al,^4^ 2002)
Male to female incidence, 1.5:1.0; 85% of patients with KD aged <5 y; peak incidence at age 9 mo (Makino et al,^10^ 2019)
3 Nationwide epidemics followed by annual increase in cases despite decreasing birth rate (Ae et al,^19^ 2020)
KD cases clustered in time and space in addition to seasonal patterns (Burns et al,^11^ 2005; Sano et al,^20^ 2016)
Distinct seasonal cycle with winter peak and fall nadir (Burns et al,^11^ 2005)
Decrease in KD incidence associated with COVID-19 pandemic (Ae et al,^17^ 2021; Hara et al,^18^ 2021)
Birth order observations suggest infants may be exposed from older, school age siblings (Namba et al,^21^ 2023)

**New findings**

Infants aged <6 mo had a distinctly different epidemiology than older children and adolescents, with little increase in case numbers over 4 decades, a different seasonal cycle with a modest summer peak, and minimal spatial coherence between prefectures
Toddlers (ages 6 to <24 mo) had a changing seasonal cycle over time, with an October peak in later years
Children and adolescents aged ≥ 3 y were the drivers of the previously described seasonal cycle. Additional peaks in the seasonal cycle in April and June may be associated with the school calendar
The seasonal cycle of KD incidence in children and adolescents aged ≥3 y was correlated across prefectures
Children aged ≥3 y had a >5-fold increase in incidence since 1990

Abbreviation: KD, Kawasaki disease.

For infants, KD incidence increased little over 1987 to 2019 and the seasonal cycle, with a summer peak, showed essentially no coherence across prefectures. It is possible that this distinct difference from the epidemiology of older children and adolescents was associated in part with the tendency of infants to be in the home. A previous analysis^21^ of birth order of patients with KD aged 6 to 18 months Japan identified having an older sibling as a risk factor associated with developing KD. The odds ratio was lower when the younger child was in day care. These observations suggest the importance of social factors in KD exposure, including person-to-person transmission from the older sibling to the younger child or increased exposure of the younger child to agents outside the home.

Toddlers exhibited a large change in their seasonal cycle in the mid-2010s in our study, which again suggests potential social or behavioral factors associated with exposure changes. At approximately that same time, the Japanese government launched a number of initiatives to provide increased day care options for families with toddlers. Most notably, the Comprehensive Support System for Children and Childcare was launched in April 2015 and expanded daycare capacity for patients younger than age 3 years (eFigure 5 and eAppendix 5 in Supplement 1). While this change in day care use does not completely explain the timing or the change in the seasonal cycle, it is plausible that this change in behavior was a contributor. Spatial correlations demonstrated that these seasonal changes in incidence among toddlers in Kyushu differed from those in other regions in Japan, suggesting that factors associated with these seasonal changes may have differed across regions.

The seasonal cycle of children and adolescents aged 3 years and older consistently exhibited minor peaks in April and June, in addition to a winter peak and an autumn nadir. Nadirs preceding secondary peaks each occurred approximately 5 weeks after the start of a school session, and there was a relative peak at the start of each school session. While it is unclear why KD incidence would decrease for the first 5 weeks of a school session, the consistent pattern suggests that levels of exposure to KD agents may differ according to times when children are in or out of school. Ae et al^22^ also found evidence for an association between school attendance and KD incidence in Japan based on the influence of COVID-19 mitigation measures. The connection to the school calendar and the decrease in cases during COVID-19 mitigation measures are both consistent with reduced exposure to aerosols that may trigger KD.

Some KD epidemiologic characteristics suggest that social or behavioral factors modulated KD transmission by person-to-person spread or other mechanisms. However, the large increase in KD incidence since 1990 and the coherence of the seasonal cycle of KD incidence in children and adolescents aged 3 years and older across most of Japan suggest waves of concurrent environmental exposures whose intensity is increasing over time and is associated with disproportionate changes in outcomes among older children and adolescents. The biologic plausibility of these 2 apparently competing mechanisms of transmission deserves consideration. One possibility is that an aerosol carried by regional scale winds harbors an infectious agent^23^ that can also be transmitted from person to person, with only individuals who are genetically susceptible manifesting KD. Infants may be exposed in the home environment through contact with older siblings. Alternatively, there could be more than 1 etiology for KD, with infants and older children and adolescents responding to different triggers.

### Limitations

We recognize that there are some limitations of the analysis presented in this study. Japan is the country of highest KD incidence, and the meticulous curation of the country’s dataset ensures that these data accurately reflect the epidemiology of KD in Japan. However, there is no standard test to diagnose KD, so the dataset relies on the clinical diagnosis by experienced physicians. There could thus exist errors in under- and over-diagnosis of KD. In addition, it is unknown if findings discussed in this study also apply to other countries.

## Conclusions

In this cross-sectional study of the historical record of KD in Japan, distinct age-specific patterns of KD incidence were revealed that suggested differences in exposures that varied by age group. First, differences in seasonal cycles and different rates of increase over the observational record between age groups suggest that exposure was modulated by social practices, including day care availability and school vacation schedules. Second, the remarkable increase and spatial coherence of KD incidence in children and adolescents aged 3 years and older suggest that environmental factors were associated with increased exposure to a KD trigger. Theories regarding the etiology of KD must account for contrasting KD temporal and spatial patterns associated with different age groups in Japan.

## Notes

Supplement 1. **eAppendix 1.** Time Series of Kawasaki Disease Incidence Rate **eFigure 1.** Time Series of Kawasaki Disease Incidence Rate by Age Group **eAppendix 2.** Significance of Differences Between Seasonal Cycles of Kawasaki Disease Incidence Over Time **eFigure 2.** Statistical Tests of Differences Between Seasonal Cycles **eAppendix 3.** Correlations of Seasonal Cycles Between Prefectures **eFigure 3.** Seasonal Cycle Correlations Between Prefectures (1988-2019) **eAppendix 4.** Changing Correlation of Seasonal Cycles Between Prefectures **eFigure 4.** Correlation of Daily Incidence Between Regions by Year **eAppendix 5.** Changing Attendance in Childcare Institutions **eFigure 5.** Number and Percentage of Children Attending Japanese Childcare Institutions

Supplement 2.
Data Sharing Statement
