# Supplementary material for: Age-Dependent Variations in Kawasaki Disease Incidence in Japan
Source: JAMA Netw Open. 2024 Feb 6;7(2):e2355001. doi: 10.1001/jamanetworkopen.2023.55001 (PMC10848069; doi:10.1001/jamanetworkopen.2023.55001)
Supplement: Supplement 1. — eAppendix 1. Time Series of Kawasaki Disease Incidence Rate eFigure 1. Time Series of Kawasaki Disease Incidence Rate by Age Group eAppendix 2. Significance of Differences Between Seasonal Cycles of Kawasaki Disease Incidence Over Time eFigure 2. Statistical Tests of Differences Between Seasonal Cycles eAppendix 3. Correlations of Seasonal Cycles Between Prefectures eFigure 3. Seasonal Cycle Correlations Between Prefectures (1988-2019) eAppendix 4. Changing Correlation of Seasonal Cycles Between Prefectures eFigure 4. Correlation of Daily Incidence Between Regions by Year eAppendix 5. Changing Attendance in Childcare Institutions eFigure 5. Number and Percentage of Children Attending Japanese Childcare Institutions [file jamanetwopen-e2355001-s001.pdf]

Supplemental Online Content

DeHaan LL, Copeland CD, Burney JA, et al. Age-dependent variations in Kawasaki disease incidence in Japan. *JAMA Netw Open*. 2024;7(2):e2355001. doi:10.1001/jamanetworkopen.2023.55001

eAppendix 1. Time Series of Kawasaki Disease Incidence Rate

eFigure 1. Time Series of Kawasaki Disease Incidence Rate by Age Group

eAppendix 2. Significance of Differences Between Seasonal Cycles of Kawasaki Disease Incidence Over Time

eFigure 2. Statistical Tests of Differences Between Seasonal Cycles

eAppendix 3. Correlations of Seasonal Cycles Between Prefectures

eFigure 3. Seasonal Cycle Correlations Between Prefectures (1988-2019)

eAppendix 4. Changing Correlation of Seasonal Cycles Between Prefectures

eFigure 4. Correlation of Daily Incidence Between Regions by Year

eAppendix 5. Changing Attendance in Childcare Institutions

eFigure 5. Number and Percentage of Children Attending Japanese Childcare Institutions

This supplemental material has been provided by the authors to give readers additional information about their work.

© 2024 DeHaan LL et al. *JAMA Network Open.*

eAppendix 1. Time Series of KD Incidence Rate

In the main body of the paper **Figure 1** shows the time series of incidence rate normalized by the rate in 1987-1992. Here, using the same data, we show the time series that has not been normalized to the 1987-1992 period (**eFigure 1**).

eFigure 1: Time Series of KD Incidence Rate by Age Group

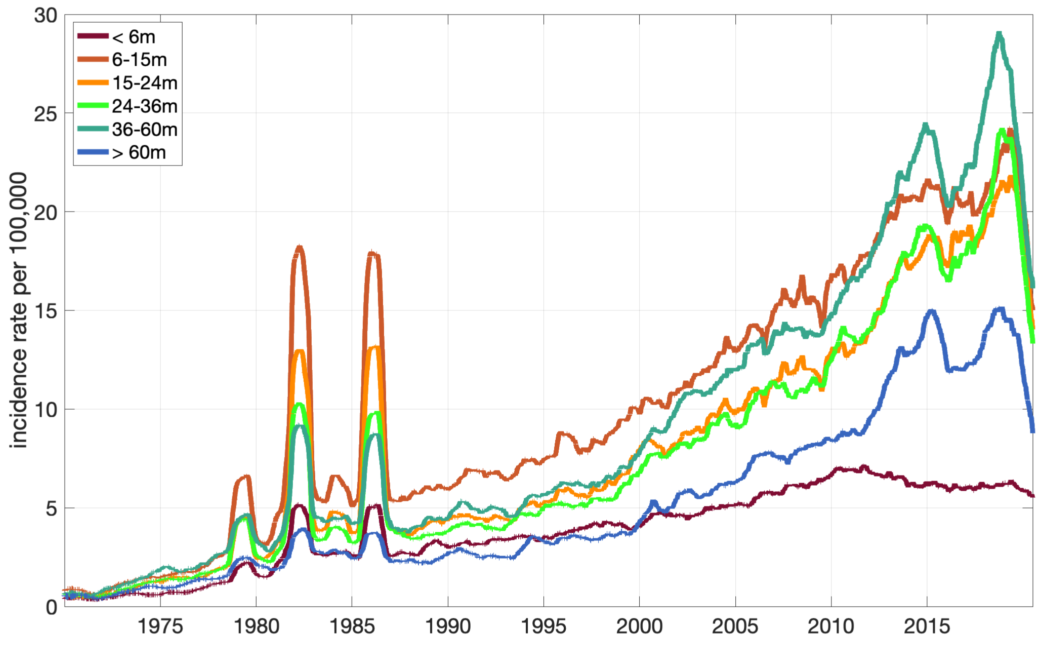

eFigure 1: Time series of KD incidence rate per 100,000 for each age group, based on daily smoothed data. The incidence rate is computed relative to the population of 0- to 14-year-olds for each year to account for the falling birth rate in Japan.

eAppendix 2. Significance of differences between seasonal cycles of KD incidence over time

To test for significant differences between seasonal cycles (**Figure 2 e-h**, main body), a random sampling approach was used to determine baseline distributions. A smoothed, normalized

© 2024 DeHaan LL et al. *JAMA Network Open.*

seasonal cycle, averaged from 1988 to 2019, was computed with a 31-day running mean. Residual values for each day of each year were found by subtracting the long-term seasonal cycle from the normalized values for each year. To construct a baseline sample ensemble, against which to compare the true seasonal cycles, sample daily series averaged over a 4-year period were constructed by averaging four residual values for each day of the year randomly selected from the 32 year observed residuals. These residuals were added to the long term smoothed seasonal cycle. This process was repeated 1000 times, yielding an envelope of 1000 sample 4-year aggregate seasonal cycles. For each month, the value from the observed seasonal cycle was ranked and compared to the distribution of the random seasonal cycles. When the observed seasonal cycle fell in the highest or lowest 2.5% of the ranking, it was considered significant at the 95% level.

eFigure 2 provides a ledger of significant changes in seasonal cycle throughout the period of record. The observed seasonal cycle (colored lines, **eFigure 2 a-d**) frequently falls outside the random sample (grey lines, **eFigure 2 a-d**). This is most notable for the toddlers in 2016-2019, but occurs in many other instances as well, demonstrating the variation in seasonal cycle over the years. Consistent with other findings presented here, the infants have very few significant changes in seasonal cycle (**eFigure 2a & 2e**). While the 2-year-olds (**eFigure 2c & 2g**) and children 3 years and older (**eFigure 2d & 2h**) have more significant changes than the infants, the largest number of significant changes happen with the toddlers (**eFigure 2b & 2f**). In the early years the toddlers had significantly lower incidence in the fall and significantly higher incidence

© 2024 DeHaan LL et al. *JAMA Network Open.*

in the spring, while in the later years there was lower incidence in the winter and much higher incidence in the fall.

© 2024 DeHaan LL et al. *JAMA Network Open.*

eFigure 2: Statistical Tests of Differences between Seasonal Cycles

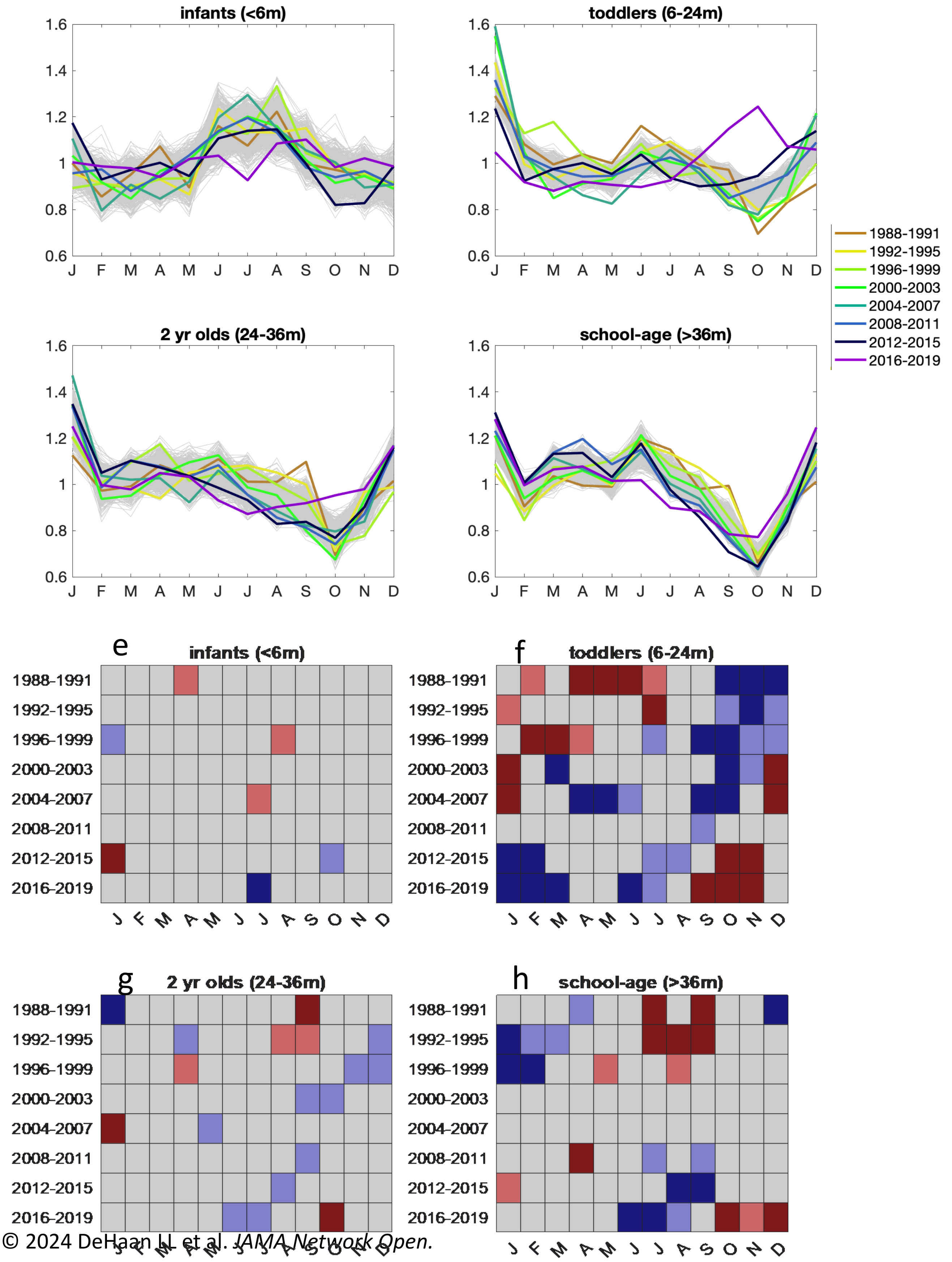

© 2024 DeHaan LL et al. *JAMA Network Open.*

eFigure 2: Panels a-d: Monthly normalized seasonal cycles of 4-year bins. Light grey lines are random sampling; colored lines are observed. Panels e-h: Significance of differences between seasonal cycles of 4-year bins (shown in panels a-d) and the 32-year average for each month of the year. Blue colors indicate significantly lower incidence; red colors indicate significantly higher incidence. Dark shades indicate that the time period exhibits a statistically different seasonal cycle for the given month at the 99% confidence level; lighter shades indicate a 95% confidence level.

eAppendix 3. Correlations of seasonal cycles between prefectures

To assess whether the seasonal cycle varied spatially across Japan, the KD historical record was disaggregated into prefectures. The seasonal cycle of each of the 47 prefectures was correlated with that of every other prefecture (**eFigure 3**). **Figure 3** of the main body shows the averages of these correlations as well as the location of each of the 47 prefectures. In the prefectures near Tokyo (prefectures 12-14) both toddlers and children 3 and older have the highest correlations between prefectures. In the southern prefectures (41-46) the toddlers do not show any significant correlation with other prefectures, while the children 3 and older have several significant correlations with prefectures across the country. Also of note is Hokkaido (prefecture #1), where the toddlers correlate more broadly with the rest of the country then the children 3 and older.

© 2024 DeHaan LL et al. *JAMA Network Open.*

eFigure 3: Seasonal Cycle Correlations between Prefectures (1988-2019)

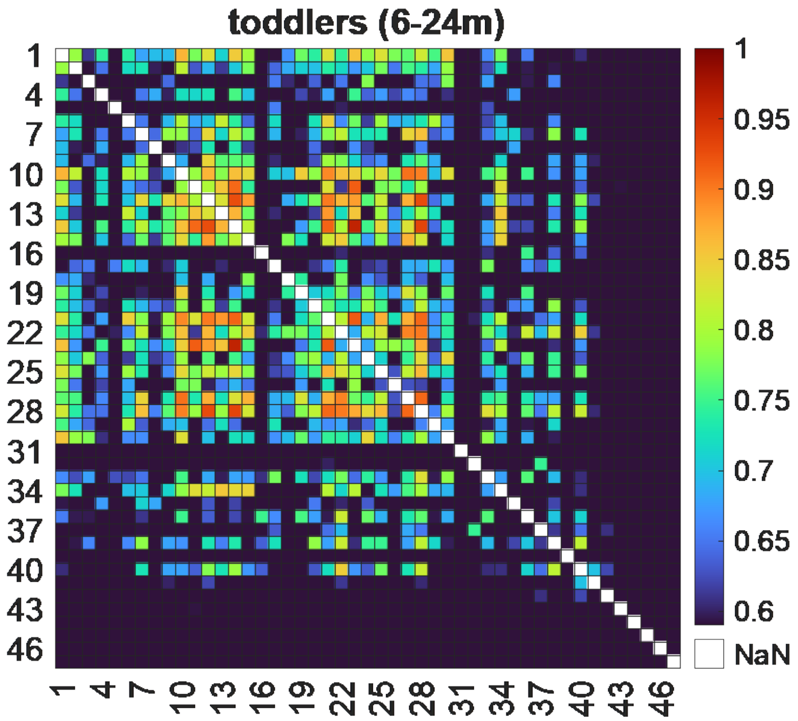

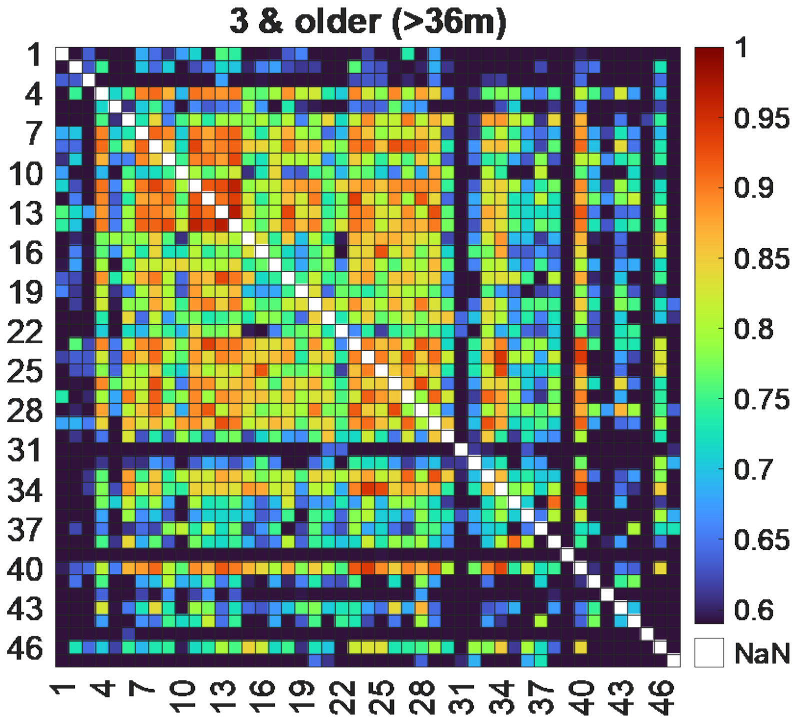

eFigure 3. Correlations of KD seasonal cycles (1988-2019) between prefectures for toddlers and children 3 and older, with values less than 0.59 (95% significance) colored dark blue. Prefectures are arranged in columns and rows from north (Hokkaido, 1) to south (Kagoshima, 46 and

© 2024 DeHaan LL et al. *JAMA Network Open.*

Okinawa, 47). See Figure 3 in the main body for maps with numbered prefectures. Note these heatmaps are symmetric.

eAppendix 4. Changing correlation of seasonal cycles between prefectures

The coherence of the seasonal cycle across prefectures changed over time for most age groups. To illustrate this, the KD incidence within the 47 prefectures were condensed into five regional subsets (**eFigure 4, right**). For each region and for each age group, the time series of the number of KD patients per day was correlated between like-timeseries from the other four regions for each year. For each age group, correlations for each prefecture were averaged across the five regions for each year to obtain a single series of correlation traversing the entire 1988-2019 period. These time varying all-Japan aggregate correlations are shown in **eFigure 4**. Data for the figure was smoothed with a 3-year running mean.

Similar to results shown in the main body, **eFigure 4** shows that infants had no statistically significant correlation between regions, and that a low level of association persisted across the entire record from 1988 to 2019. In sharp contrast, two-year-olds and children 3 years and older experienced increasing correlations in the past two decades, rising to an average of 0.6 or greater by 2019. Toddlers exhibited a more complicated pattern, wherein correlation between regions also increased in the early 2000s, but then dropped starting around 2010 and began to rebound after 2015. The reduction in correlation in 2010 coincides with the large shift in the seasonal cycle of countrywide KD incidence shown in **Figure 2f** of the main body. Increased correlation of regional toddler incidence after 2015 is somehow associated with a more widespread shift in the seasonal cycle to have a reduced peak in KD incidence in winter and

© 2024 DeHaan LL et al. *JAMA Network Open.*

increased incidence in autumn KD incidence. To test the robustness of the features shown in **eFigure 4,** this computation was repeated (not shown) using differing numbers of regions and different smoothing applied to the data. While the exact values differ with the number of regions and the level of smoothing, the age-related correlation structure obtained in **eFigure 4** here are common to all variations of the calculation.

eFigure 4: Correlation of Daily Incidence between Regions by Year

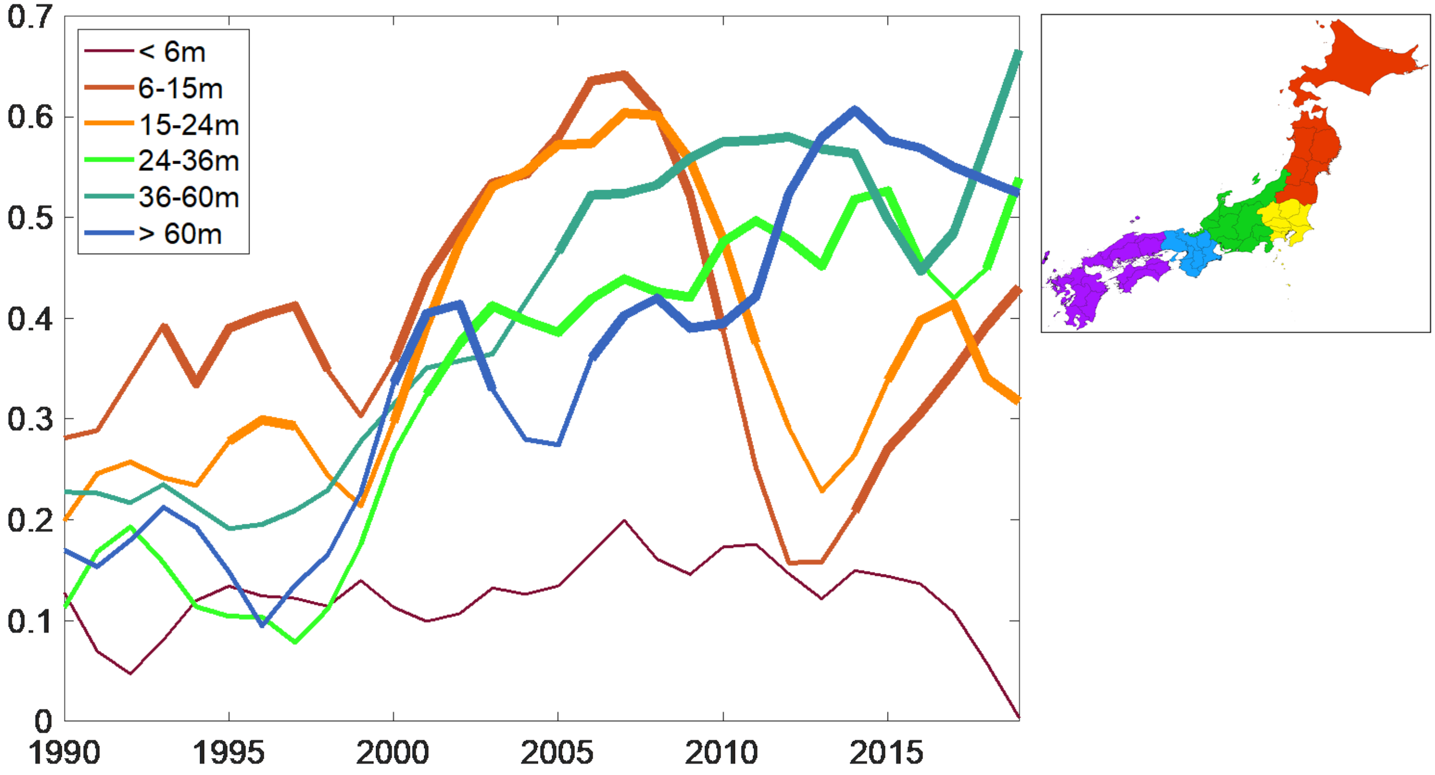

eFigure 4. Correlation of daily incidence between each of five regions of Japan (shown right) and every other region. Correlation is computed for each year individually and averaged over the five regions. Thick lines indicate at least one pair of regions had a correlation significant at 95% (above 0.59).

© 2024 DeHaan LL et al. *JAMA Network Open.*

eAppendix 5. Changing attendance in childcare institutions

The number of children in daycare institutions in Japan has steadily increased for the last two decades, with a significant increase in institutions in 2015, as shown in **eFigure 5**.

eFigure 5: Number and Percent of Children Attending Japanese Childcare Institutions

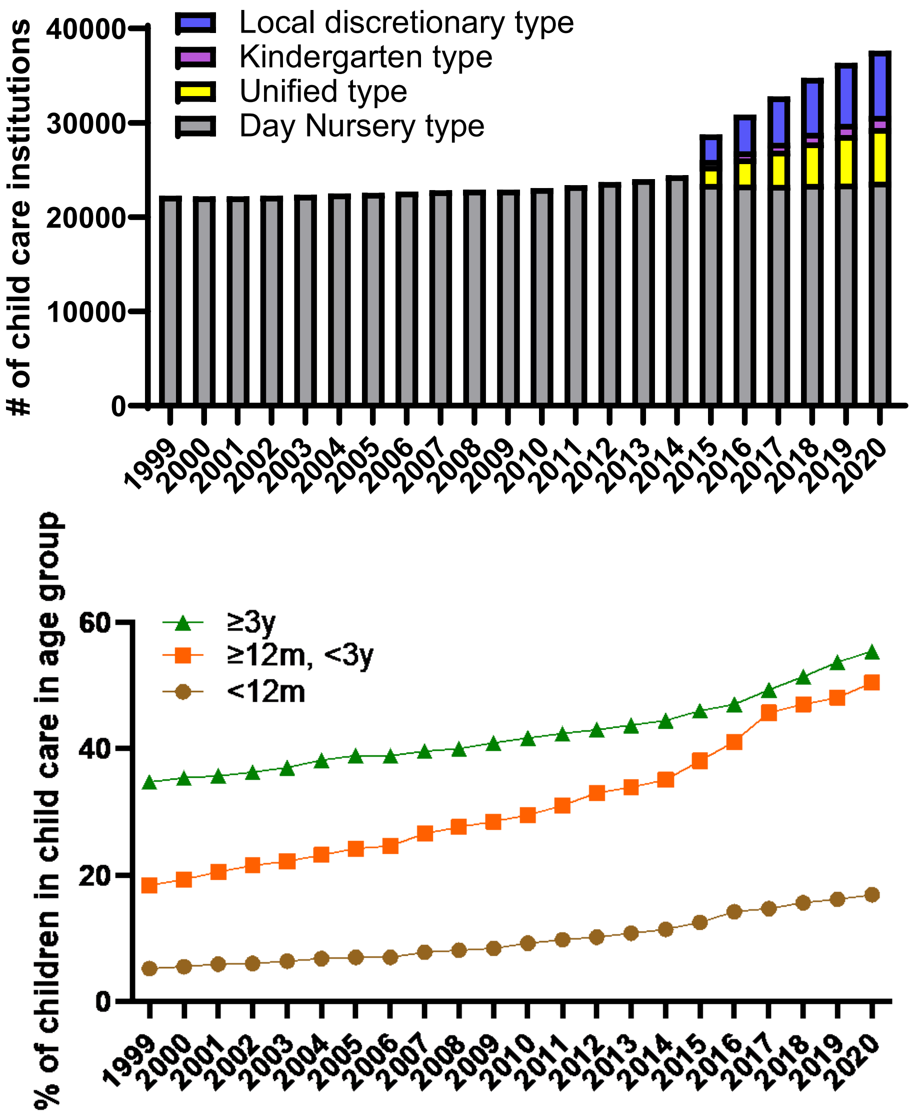

eFigure 5: Number of childcare institutions of various types since 1999 (top). Percent of children in childcare institutions (bottom). Local discretionary type: 0-2-year-old, Kindergarten type: 3-5-year-old, Unified type: 0-5-year-old, Day nursery type: 0-5-year-old. Data from the

© 2024 DeHaan LL et al. *JAMA Network Open.*

reports (2009-2020) by Ministry of Health, Labor, and Welfare:
https://www.mhlw.go.jp/stf/newpage_27446.html)

© 2024 DeHaan LL et al. *JAMA Network Open.*
