# Supplementary material for: Age-Dependent Variations in Kawasaki Disease Incidence in Japan
Source: JAMA Netw Open. 2024 Feb 6;7(2):e2355001. doi: 10.1001/jamanetworkopen.2023.55001 (PMC10848069; doi:10.1001/jamanetworkopen.2023.55001)
Supplement: Supplement 2. — Data Sharing Statement [file jamanetwopen-e2355001-s002.pdf]

Data Sharing Statement

DeHaan. Age-Dependent Variations in Kawasaki Disease Incidence in Japan. *JAMA Netw Open*. Published online February 6, 2024. doi:10.1001/jamanetworkopen.2023.55001

Data
Data available: No

Additional Information
Explanation for why data not available: The Japanese database is under the jurisdiction of Jichi University in Japan and interested parties should contact Dr. Yosikazu Nakamura.
